# Maxillary sinus textiloma: a case report

**DOI:** 10.1186/1752-1947-4-288

**Published:** 2010-08-24

**Authors:** Yoann Pons, Thomas Schouman

**Affiliations:** 1 Maxillofacial Surgery Department, AP-HP - Pitié-Salpêtrière University Hospital, University of Paris 6, France

## Abstract

**Introduction:**

Textilomas have been reported in many locations. We report the first case of textiloma located in the maxillary sinus that mimicked a sinus cyst recurrence on computed tomography images.

**Case presentation:**

A 60-year-old Caucasian man was referred for persistent infection of the right maxillary sinus. A maxillary sinus benign cyst had been removed three months before. Computed tomography showed a sinus opacity evoking a cyst recurrence. A new operation was planned to remove the cyst by a Caldwell-Luc approach. After excision of very thick fibrous tissue, a compress was discovered in the maxillary sinus. The patient did not present with any sinus infection after the operation.

**Conclusion:**

The surgeon should always take into account the possibility of textilomas in a patient with a history of sinus surgery.

## Introduction

Textiloma can be defined as a mass within the body composed of cotton matrix, which usually refers to a retained surgical sponge or compress, surrounded by a foreign-body reaction [1].

Most cases of textiloma reported in the literature have been connected to abdominal, orthopaedic and cardiothoracic surgery [1-3]. At the head level, few intra-cranial cases have been reported [4,5]. No case, to date, has been reported at the face level. The authors reported the first case of textiloma located in the maxillary sinus.

## Case presentation

**Figure 1:**
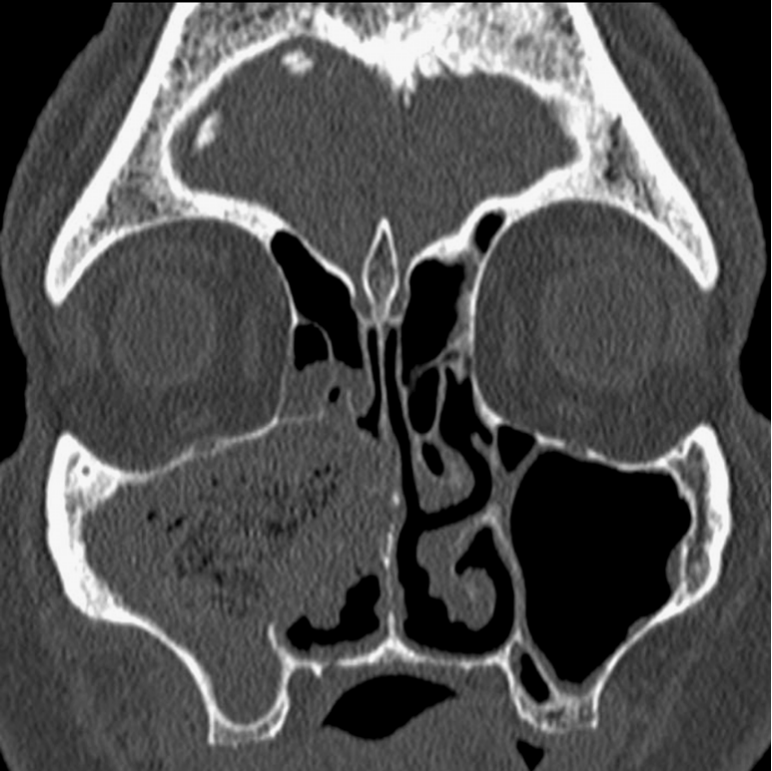
**CT scan imaging showing the textiloma located in the maxillary sinus and mimicking a cyst recurrence**.

**Figure 2:**
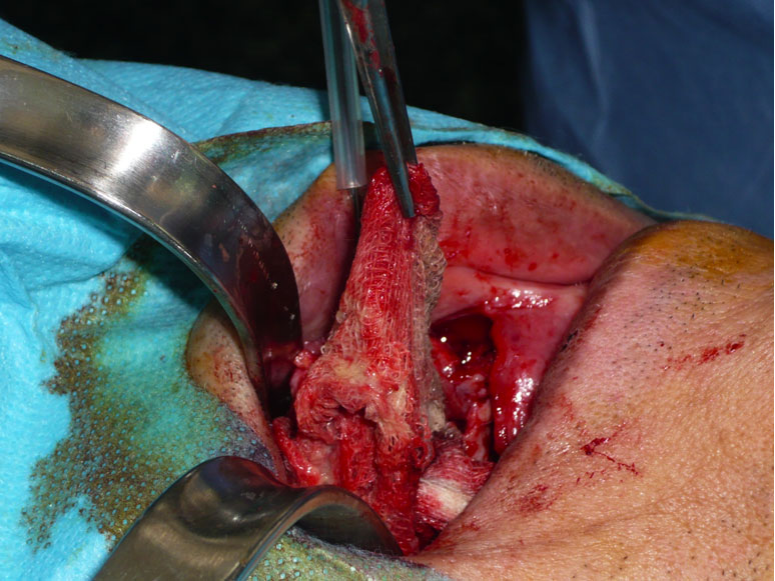
**Operative view of the compress extraction from the maxillary sinus**.

A 60-year-old Caucasian man was referred to us for persistent infection of the right maxillary sinus. He was operated on three months ago for a benign cyst. A Caldwell-Luc operation was performed. Since this operation, the patient complained of having recurrent sinusalgia with purulent rhinorrhea. Computed tomography (CT) showed a sinus opacity evocating a cyst recurrence (Figure 1). A new surgery was planned to remove the cyst by a new Caldwell-Luc operation. After excision of very thick fibrous tissue, a compress was discovered in the maxillary sinus (Figure 2). The patient did not present any sinus infection after the operation.

## Conclusions

The main complication of a maxillary sinus textiloma is a persistent infection. In this case, the sinusitis was limited (that is, no orbital or meningeal complications occurred).

The erroneous diagnosis of a mass provoked by the presence of a textiloma was frequently reported in the literature in other regions [1-5]. In this case, both the radiologist and the surgeon had suggested the diagnosis of cyst recurrence, given the CT-scan examination. However, at a second viewing of the images, some radiologic signs of textiloma were noticed: the mass was heterogeneous with a rectilinear alternation of thin bands (solid-band and air-band densities) that corresponded to the meshing of the compress. Moreover, foreign bodies of the maxillary sinus are a common cause of persistent infection. The diagnosis was finally corrected by the surgery, which definitively cured the patient.

The suspicion of textiloma should be raised when a patient with a history of previous maxillary sinus surgery presents with a history of chronic sinus infection associated with a sinus mass on CT images, even though textiloma is unlikely to be found in such a small cavity.

## Consent

Written informed consent was obtained from the patient for publication of this case report and accompanying images. A copy of the written consent is available for review by the Editor-in-Chief of this journal.

## Competing interests

The authors declare that they have no competing interests.

## Authors' contributions

YP redacted the manuscript. TS supervised the manuscript. Both authors read and approved the final manuscript.

## Authors' information

The authors are two medical doctors. Yoann Pons is a head and neck surgeon.

Thomas Schouman is a maxillofacial surgeon.
